# Prevalence of Asymptomatic* Mycobacterium tuberculosis* Infection in Charcoal Producers: A Cross-Sectional Study in Kaase, Ghana

**DOI:** 10.1155/2018/9094803

**Published:** 2018-08-02

**Authors:** Benjamin Kwame Senya, Nketiah Bernard Anim, Bright Segu Kobena Domson, Patrick Adu

**Affiliations:** Department of Medical Laboratory Science, College of Health and Allied Health Sciences, University of Cape Coast, Ghana

## Abstract

**Background:**

Charcoal production is a significant economic activity in Ghana. However, there is scarcity of data on the risk of acquiring* Mycobacterium tuberculosis* infection among charcoal producers in Ghana, even though persistent smoke exposure is a known predisposition factor.

**Methods:**

This cross-sectional study recruited 40 charcoal producers: 6 males and 34 females. Two sets of early morning sputum samples were collected from each participant and examined for the presence of acid-fast bacilli (AFB) using fluorescent microscopy. Structured questionnaires were used to retrieve demographic data from each participant. Data were analyzed using SPSS version 21 and presented as frequencies and proportions. Categorical variables were compared using Chi-square test. Significant difference was identified as *p* < 0.05 at 95% confidence interval.

**Results:**

Overall, 2/40 (5%) of the participants demonstrated AFB in their sputum. All participants with AFB positive sputum were females and had 6–10 years of experience in charcoal production. Whereas coughing was the most self-reported symptom by the charcoal producers, none complained of blood in sputum. Also, only 9/40 (22.5%) had knowledge about the* Mycobacterium tuberculosis*-infection risk associated with charcoal production. Moreover, 62.5% (25/40) of participants had no formal education.

**Conclusion:**

Education on personal protection equipment must be a public health priority in these charcoal producers in Ghana as sawdust and smoke exposure may predispose charcoal producers to acquisition of tuberculosis.

## 1. Introduction

Tuberculosis (TB) is a global public health problem caused by* Mycobacterium tuberculosis*. One-third of the world's population is thought to be infected with TB [1]. According to Patra et al. [2], about 8.6 million people developed active TB with at least 1.3 million mortalities mainly in resource-limited countries. In 2014, there were 9.6 million cases of active TB which resulted in 1.5 million deaths with more than 95% of the deaths occurring in developing countries [1]. Also, the number of cases of TB per 100,000 people was highest in sub-Saharan Africa in 2007 [3]. TB is among the commonest communicable diseases in Ghana with a reportedly over 250,000 more Ghanaians acquiring* M. tuberculosis* infection each year [4].

TB is transmitted by droplet inhalation produced by coughing, singing, and sneezing of an infected person. Certain lifestyle choices and occupations are known to predispose individuals to risk of contracting the bacterium and subsequently developing active infection. Previous findings have shown that exposure to particulate matter and smoke from carbonizing wood is a risk factor for developing TB in case-control studies conducted in India [5], Nepal [6], and Metropolitan Mexico City [7]. Another study also showed that exposure to wood smoke is associated with the development of TB in children aged ≤17 due to the immaturity of their respiratory and immune system [8]. Also, analysis of data from 200,000 Indian adults found an association between self-reported tuberculosis and exposure to wood smoke [9].

The global production of wood charcoal was estimated at 47 million metric tonnes in 2009 and increased by 9% since 2004 with Africa accounting for 63% of global production. Ghana is rated as the 9th producer of wood charcoal, producing 3% of the entire world wood charcoal [10]. Even though charcoal production is a significant occupation in Ghana, studies assessing the incidence of asymptomatic TB infection among the charcoal producers are nonexistent. This study thus sought to provide such empirical data. 

## 2. Materials and Methods

### 2.1. Study Design/Population

This was a cross-sectional study based on convenience sampling that recruited charcoal production workers with a minimum of one-year work experience and at least 15 years old at Kaase, a suburb of Kumasi, the regional capital of the Ashanti Region, Ghana. A total of 40 participants (6 males and 34 females) who gave informed consent were recruited.

### 2.2. Data Collection

Two (2) sets of morning sputum specimens (3-5 ml each) at 30 minutes intervals were collected from each participant for the study since sputum is best collected in the morning soon after the patient wakes up and before any antiseptic mouth-wash use.

### 2.3. Ethical Consideration

The study had approval from the University of Cape Coast Institutional Review Board** (UCCIRB/CHAS/2016/47)** and the Kumasi South Hospital authorities. A written consent was sought from interested participants after the procedures were clearly explained to them. Results and records were strictly kept confidential.

### 2.4. Sputum AFB

Sputum specimen from each participant was identified with a unique numbering system followed by A or B indicating soon after the patient wakes up and before any antiseptic mouth-wash use, respectively. Florescence microscopy was done after fluorochrome staining following standard protocols and bacteria found graded according to the International Union against Tuberculosis and Lung Disease (IUATLD)/WHO for reporting the sputum smear microscopy results [11].

### 2.5. Data Analysis

The data obtained was entered and stored in Microsoft Excel and analyzed using Statistical Package for Social Sciences (SPSS) software version 21.0 (IBM Inc., USA). Summary descriptive statistics were used to describe the study population and reported using frequency table, bar chart, and cross-tabulation. Pearson's Chi-square model (*χ*^2^) was used where appropriate to compare categorical variables between groups. Significant differences were identified as *p*-value < 0.05 at 95% confidence interval.

## 3. Results

The demographic characteristics of the participants are shown in Table 1. Among these, 15.0% were males and 85.0% were females. Majority of the males were within the age groups 15 to 24 years and 35 to 44 years whereas most of the females were within the age group 35 to 44 years.

The knowledge of the participants about TB is shown in Table 2. Whereas 2/6 (33.3%) of males had some knowledge about TB, only 7/34 (20.6%) females had knowledge about TB. Overall, only 22.5% (9/40) of the participants had knowledge about TB. Also, whereas most [3/8 (37.5%)] of the participants who had knowledge about TB were within the age group 25–34 years, only 12.5% (1/8) of participants within the age group of 45–54 years had knowledge about TB. Additionally, 62.5% (25/40) of the participants had had no formal education. Whereas 20% of participants with no formal education had some knowledge on the TB-risk associated with charcoal production, none of participants with junior high education had any such knowledge.

**Figure 1:**
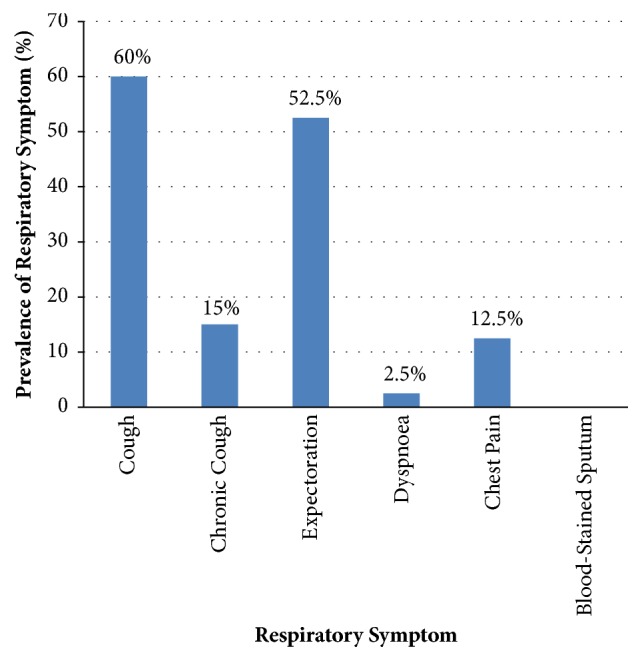
**Self-reported prevalence of respiratory symptoms among participants. **Chronic cough refers to any persistent cough lasting more than four weeks.

Figure 1 presents self-reported prevalence of respiratory symptoms among the 40 participants. The most prevalent self-reported respiratory symptoms in the participants were cough (60.0%) and expectoration (52.5%), with none reporting of blood-stained sputum (0.0%).

Table 3 presents the grading of AFB in sputum smear specimen as evaluated by fluorescent microscopy. Overall, 5.0% (2/40) of the participants tested positive to AFB; the grading was >scanty but less than 3+.

All the participants with positive AFB sputum smear were females (Table 4). Additionally, all female participants with positive AFB sputum smear had 6–10 years of experience with charcoal production.

## 4. Discussion

Studies have shown that combustion of wood releases large particulate matter such as carbon monoxide, nitrogen oxide, formaldehyde, and polyaromatic hydrocarbons which can deposit deep into the alveoli and can cause considerable damage to the respiratory system predisposing those exposed to such smoke to TB infection [12, 13]. Although charcoal production workers abound in Ghana, there has not been any systematic study to address the long-term health implication of this vocation. Within the limits of the fluorescent microscopy technique, our study found 5% prevalence of asymptomatic TB infection in charcoal producers in the Kaase community in Kumasi.

Our reported 5% prevalence of asymptomatic* Mycobacterium tuberculosis* infection is higher than the 1% prevalence reported in a similar cross-sectional study in India [14]. Our study is in agreement with previous reports that found wood smoke exposure as an important risk factor for the development of TB [2, 5–8, 15]. Interestingly, all the participants with positive AFB smear in the present study were females. This is not surprising because unlike the male participants whose job were only to pack the wood, the females, in addition to packing the wood, were also responsible for covering the pile with sawdust as well as controlling the carbonization process. Therefore, the females in our study population were exposed to larger quantities of smoke particles and sawdust, thus making them prone to TB infection. This notwithstanding, the male participants were considerably fewer than the females in the present study and this could have also skewed the finding of only females being infected with the bacterium. All the 2 (5%) infected were workers who had been in the business for about 6–10 years.

The most prevalent self-reported respiratory symptom in this study was cough (60.0%) followed by expectoration (52.5%) with none of the participants reporting blood in their sputum. Saw dust used to cover the packed wood could have caused the inhalation of sawdust in addition to smoke particles which may irritate the respiratory system triggering the cough and expectoration reported by the participants. This is in agreement with a previous study in Brazil that reported cough as the most common lower respiratory tract symptom among charcoal production workers [16]. However, whereas some participants in the study in Brazil smoked, none of the participants in the present study smoked. It is important to note that none of the participants in the present study used personal protection equipment (PPE) when undertaking their charcoal production. This might have increased the exposure to smoke and particulate matter in the participants in the present study.

The limitations of the present study include the small sample size used as well as the fluorescent microscopy technique employed. Whereas a larger sample size would have increased the statistical power, we anticipate that polymerase chain reaction-based technique such as GeneXpert could have estimated a higher prevalence considering its higher sensitivity.

## 5. Conclusion

Education on personal protection equipment must be a public health priority in these charcoal producers in Ghana as sawdust and smoke exposure may predispose charcoal producers to acquisition of tuberculosis.

## Figures and Tables

**Table 1:** Age group of participants according to gender of participants.

**Characteristic**	**Male (**%**)**	**Female (**%**)**	**Total (**%**)**
**Age Group**			
15-24	2 (5.0)	3 (7.5)	5 (12.5)
25-34	1 (2.5)	7 (17.5)	8 (20.0)
35-44	2 (5.0)	9 (22.5)	11 (27.5)
45-54	1 (2.5)	7 (17.5)	8 (20.0)
55-64	0 (0.0)	3 (7.5)	3 (7.5)
≥65	0 (0.0)	5 (12.5)	5 (12.5)

**Table 2:** Knowledge of TB-infection risk associated with charcoal production.

	**TB Knowledge**	
	**Yes**	**No**
**Gender**		
Male	2	4
Female	7	27
**Age Group**		
15-24	1	4
25-34	3	5
35-44	2	9
45-54	1	7
55-64	1	2
≥65	1	4
**Educational Level**		
Primary	3	4
JHS	0	5
SHS	1	2
None	5	20

**Table 3:** Grading of AFB in smears using fluorescent microscopy.

**Grade of AFB**	**Number of Participants**	**Percentage**
	**(N=40)**	**(%)**
**Negative**	38	95.0
**Scanty**	0	0.0
**1+**	1	2.5
**2+**	1	2.5
**3+**	0	0.0

**Table 4:** Prevalence of TB among participants based on gender and number of years of charcoal production.

	**TB Status**	
	**Positive **	**Negative **
	**N (**%**)**	**N (**%**)**
**Gender**		
Male	0 (0.0)	6 (15.0)
Female	2 (5.0)	32 (80.0)
**Years of work**		
1-5	0 (0.0)	15 (37.5)
6-10	2 (5.0)	19 (47.5)
>10	0 (0.0)	4 (10.0)

## Data Availability

The data used to support the findings of this study are available from the corresponding author upon request.
